# Comprehensive analysis of lncRNA expression profiles and identification of functional lncRNAs in lung adenocarcinoma

**DOI:** 10.18632/oncotarget.7559

**Published:** 2016-02-21

**Authors:** Mantang Qiu, Dongjie Feng, Haitian Zhang, Wenjia Xia, Youtao Xu, Jie Wang, Gaochao Dong, Yewei Zhang, Rong Yin, Lin Xu

**Affiliations:** ^1^ Department of Thoracic Surgery, Nanjing Medical University Affiliated Cancer Hospital, Jiangsu Key Laboratory of Molecular and Translational Cancer Research, Cancer Institute of Jiangsu Province, Nanjing, China; ^2^ The Fourth Clinical College of Nanjing Medical University, Nanjing, China; ^3^ The First Clinical Medical College of Nanjing Medical University, Nanjing, China; ^4^ Department of Scientific Research, Nanjing Medical University Affiliated Cancer Hospital, Cancer Institute of Jiangsu Province, Nanjing, China; ^5^ Department of General Surgery, Nanjing Medical University Affiliated Cancer Hospital, Cancer Institute of Jiangsu Province, Nanjing, China

**Keywords:** lung adenocarcinoma, lncRNA, expression profile, LCAL6

## Abstract

Increasing evidence has highlighted the critical roles of long non-coding RNAs (lncRNAs) as biomarkers and therapeutic targets for cancer. Here, we characterized lncRNA expression profile in lung adenocarcinoma (LUAD). A training-validation method was applied to identify differentially expressed lncRNAs between LUAD samples and normal samples. 856 differentially expressed lncRNAs were identified. Bioinformatics analyses showed that these lncRNAs were located nearby transcription start sites and key regulators of cancer and these lncRNAs were involved in many critical biological processes. We found the lung cancer associated lncRNA 6 (LCAL6) was significantly unregulated and predicted survival in LUAD. Silence of LCAL6 inhibited LUAD tumor cell growth both *in vitro* and *in vivo*. To summary, we comprehensively analyze lncRNA expression profile in LUAD and provide resources for further search for clinical biomarkers and therapeutic targets of LUAD.

## INTRODUCTION

Lung cancer is the most common kind of malignant tumors and non-small cell lung cancer (NSCLC) accounts for more than 80% newly diagnosed cases of lung cancer [1]. According to the WHO classification, lung adenocarcinoma (LUAD) and lung squamous cell cancer (LSCC) are the two major histological types of NSCLC [2]. The incidence of LUAD is increasing recent years and LUAD has been the most common histological type of lung cancer [3]. Despite the advantages achieved in the treatment of LUAD, the prognosis of LUAD is still poor since most LUAD patients are at advanced stage when diagnosed [4, 5]. Thus, it is urgent to identify sensitive biomarker for early diagnosis and discover the underlying molecular mechanism of LUAD [6].

Long noncoding RNAs (lncRNA) are RNA transcripts larger than 200nt without protein coding capacity [7, 8]. According to the proximity to nearby coding genes, lncRNAs could be categorized to 5 classes: sense, antisense, intergenic, bidirectional and intronic [9]. It has been proved that lncRNAs function as important roles in initiation and progression of cancer. Several cancer-associated lncRNAs have been proved as biomarkers and therapeutic targets for cancer [10-12]. Characterization of aberrantly expressed molecular markers has been utilized to identify molecular biomarkers and elucidate the mechanisms of tumor progression and metastasis [13, 14]. Compared with protein-coding genes, lncRNAs are also actively transcribed and lncRNAs have a more spatially and temporally dependent expression pattern [15]. Herein, lncRNA expression profiles may help identify key lncRNAs involved in LUAD carcinogenesis. Compared with RNA sequencing, reannotation and profiling lncRNA expression from published microarray datasets is of low cost and feasible and it has been applied by many researchers [13, 16].

In this study, we identify differentially expressed lncRNAs between LUAD and normal lung tissues by analyzing 215 LUAD and 164 normal lung tissue samples and reannotation of Affymetrix HG-U133Plus 2.0 arrays. 856 differentially expressed lncRNAs are identified and we find the lncRNA, lung cancer associated transcript 6 (LCAL6) is upregulated and promotes tumor progression in LUAD.

## RESULTS

### Microarray datasets characteristics

**Figure 1:**
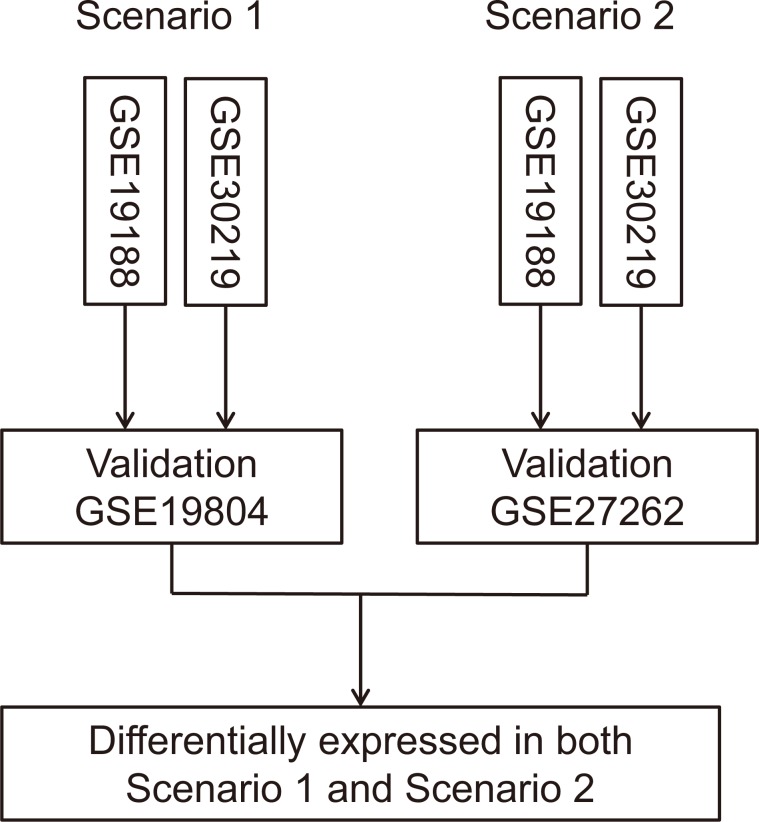
Flow chart of data analysis process The larger datasets (GSE19188 and GSE30219) were used as training sets, and the smaller datasets (GSE19804 and GSE27262) were treated as the validation sets to improve statistical power.

Four eligible microarray datasets were identified from the GEO database: GSE19188 [17], GSE19804 [18], GSE27262 [19], and GSE32901 [20]. Basic characteristics of 4 datasets were shown in Table 1. The larger datasets (GSE19188 and GSE30219) were used as training sets, and the smaller datasets (GSE19804 and GSE27262) were treated as the validation sets (Figure 1), as it has been shown that the proportion of misclassifications decreases as the number of samples in the training set increases [21].

**Table 1:** Characteristics of the 4 eligible datasets analyzed

Dataset	References	Num of LUAD	Num of normal	TNM stage
GSE19188	Hou J, et al; 2010	45	65	I
GSE19804	Lu T, et al; 2010	60	60	I-IV
GSE27262	Wei T, et al; 2012	25	25	I
GSE30219	Rousseaux S, et al, 2013	85	15	I-IV

### Differential lncRNA expression profiles in LUAD

**Figure 2:**
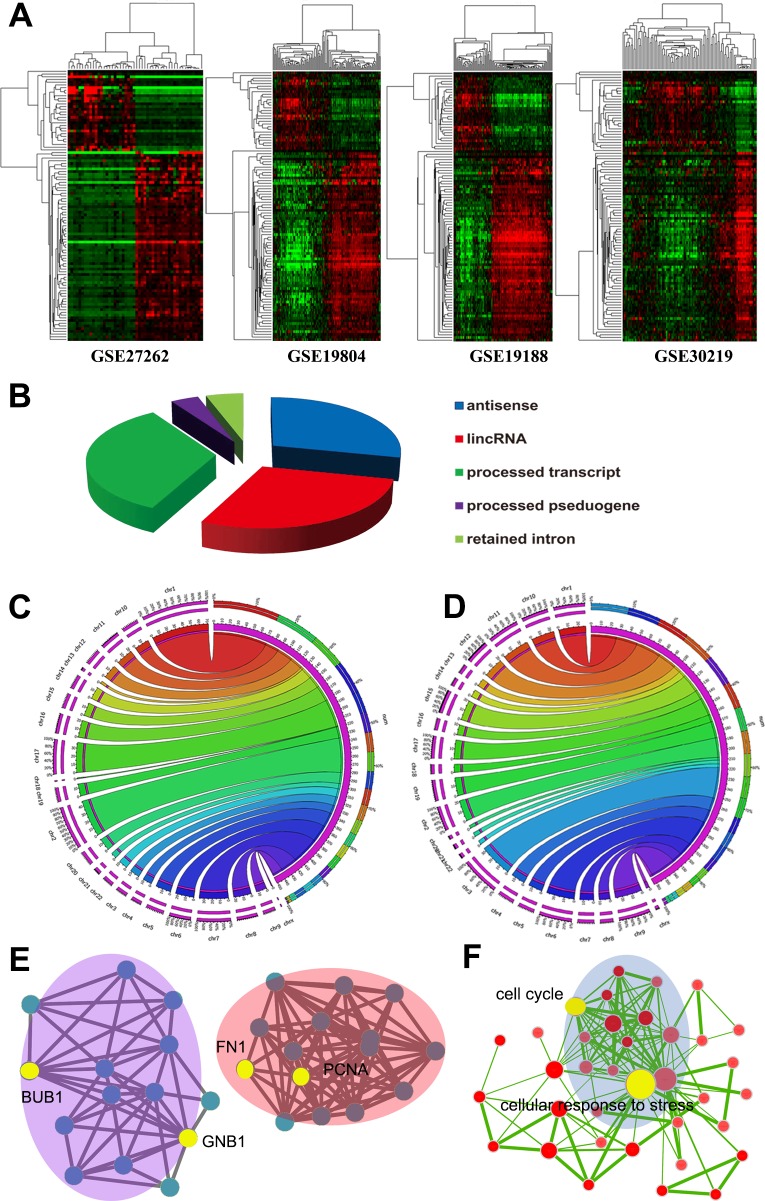
Expression heapmap of lncRNAs in the 4 datasets **A.** The left panels are LUAD samples and right panels are normal samples. Differentially expressed lncRNAs showed similar expression pattern in the 4 datasets. Biotypes of differentially expressed lncRNAs identified **B.** Distribution of upregulated lncRNAs **C.** and downregulated lncRNAs **D.** according to chromosome location. GREAT analyses found many differential lncRNAs are located nearby important cancer associated coding genes **E.** and they may be involved in various biological processes **F.** Each node indicates a cancer associated protein-coding gene **E.** of a GO biological process item **F.**

Although several LUAD associated lncRNAs have been characterized [22, 23], the comprehensive landscape of differentially expressed lncRNAs remains unknown. In the current study, Affymetrix HG-U133Plus 2.0 microarray probe sets were reannotated and 8068 probe sets mapped to lncRNAs were achieved (Supplementary Table S1). Then, we analyzed differentially expressed lncRNAs between LUAD and normal tissues using a training-validation approach and 856 differentially expressed lncRNAs were identified (Supplementary Table S2). Several characterized cancer-associated lncRNAs were identified, such as PVT1 [24], TINCR [25], and GAS5 [26]; while the well-known lung cancer-associated lncRNA, MALAT1 [27] was not included. 100 top differentially expressed lncRNAs were extracted and the heatmaps showed that lncRNAs expression patterns of the 4 eligible datasets were highly concordant, reflecting a high consistence in expression patterns of these lncRNAs among different datasets (Figure 2A). 4 lncRNAs were also selected and validated by qRT-PCR in 20 pairs of lung cancer tissues and adjacent tissues (Supplementary Figure S3). Expression patterns of all 4 lncRNAs were consistent with our analysis results, showing the analysis results were solid.

As shown, antisense, large intergenic noncoding RNA (lincRNA), and processed transcripts are 3 most common biotypes of lncRNAs (Figure 2B). According to chromosomal location, most up-regulated lncRNAs were located in chromosomes 1, 2, 17, and 7; while chromosomes 3, 6, 1,9, and 11 harbored most down-regulated lncRNAs (Figure 2C, 2D). To explore the biological function of the differentially expressed lncRNAs we identified, the genomic regions enrichment of annotations tool (GREAT) [28] was used to predict their biological functions. Most differential lncRNAs are located near transcription start sites, since it has been proved that lncRNAs are actively transcribed from human genome (Supplementary Figure S4). We discovered that many lncRNAs were located nearby important key regulators of lung cancer, including FN1, PCNA, BUB1, GNB1, and other cancer associated genes (Figure 2E). The proximity of lncRNAs to protein-coding genes indicating those lncRNAs might potentially regulat nearby protein-coding genes *in cis*. Based the genomic region of lncRNAs and their neighbor coding genes, GREAT analysis predicted that these lncRNAs may be involved in various vital biological processes, such as cell cycle, cellular response to stress, DNA repair, apoptosis, induction of programmed cell death (Figure 2F).

### LncRNA expression profiles provide resources to identify functional lncRNAs in LUAD

**Figure 3:**
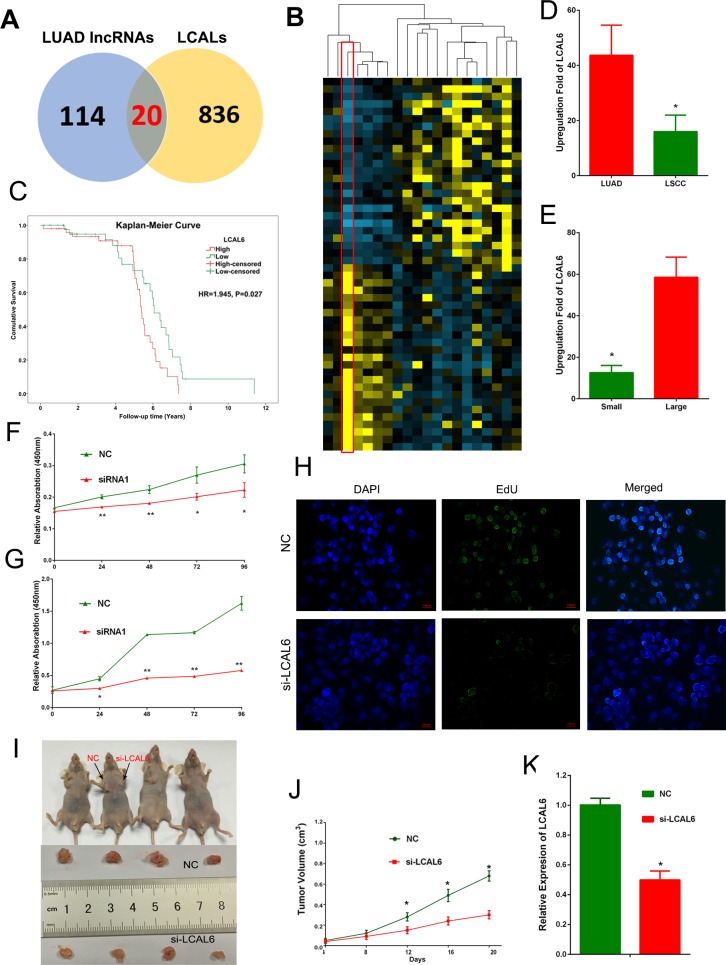
20 lncRNAs were shared by our analyses and LCALs **A.** Expression heatmap of the 20 lncRNAs in the GSE27262 dataset **B.** High expression level of LCAL6 indicates poor prognosis of LUAD patients (C, HR = 1.945, 95% CI: 1.081-3.501, *P* = 0.027,). In an expression cohort of NSCLC patients, LCAL6 level was higher **D.** in LUAD compared with LSCC and higher in patients with larger tumor size **E.** CCK8 assay (F, A549 cells; G, H1299 cells) and EdU (H, A549 cell) assay showed that silence of LCAL6 by siRNA inhibited LUAD cells proliferation. In the xenograft tumor models, silence of LCAL6 inhibited xenograft tumor growth *in vivo* (I);J, tumor volume; K: tumor weight.

Using lncRNA expression profiles as a primary filter, we might be able to generate a reliable and clinically relevant lncRNAs for scanning of functional lncRNAs in LUAD. In addition to microarray data, White et al has analyzed lncRNA expression profile using RNA-seq data and identified hundreds of lung cancer associated lncRNAs (LCALs) [29]. We used the LCAL lists to narrow down the 856 lncRNAs signature and yielded 20 lncRNAs (Supplementary Table S5). Expression heatmap of the 20 lncRNAs in GSE27262 dataset was shown in Figure 3. Then, we analyzed the prognostic value of these lncRNAs in an independent cohort of LUAD samples (GSE50081). High expression level of BLACAT1 (also named as LCAL6) indicated poor survival of LUAD patients (HR = 1.945, 95% CI: 1.081-3.501, *P* = 0.027, Figure 3). In another cohort of 67 lung cancer patients, we found expression level of LCAL6 was higher in LUAD than LSCC (*P* = 0.014) and correlated with larger tumor size (*P* < 0.01).

LCAL6 is a 2616nt antisense lncRNA, located in the chromosome region of 1q32.1. To probe the potential role of LCAL6 in LUAD, we firstly designed small interfering RNAs (siRNAs) that specifically targeted and depleted LUAD (Supplementary Figure S6). CCK8 and EdU assay showed that after depletion of LCAL6, proliferation ability of A549 (Figure 3) and H1299 (Supplementary Figure S6) cells was significantly inhibited. We next developed xenograft tumor models using A549 cells transfected with negative control (NC) siRNA or siRNA targeting LCAL6. As expected, xenograft tumors growth was inhibited in the si-LCAL6 group and the tumor volume and tumor weight were lower than that of the NC group (Figure 3). The staining of Ki67, a proliferation marker was also weaker in tumor tissues derived from A549 cells transfected with si-LCAL6, convincing the inhibition of proliferation (Supplementary Figure S6). Thus, silence of LCAL6 also inhibited LUAD growth both *in vitro* and *in vivo*.

**Figure 4:**
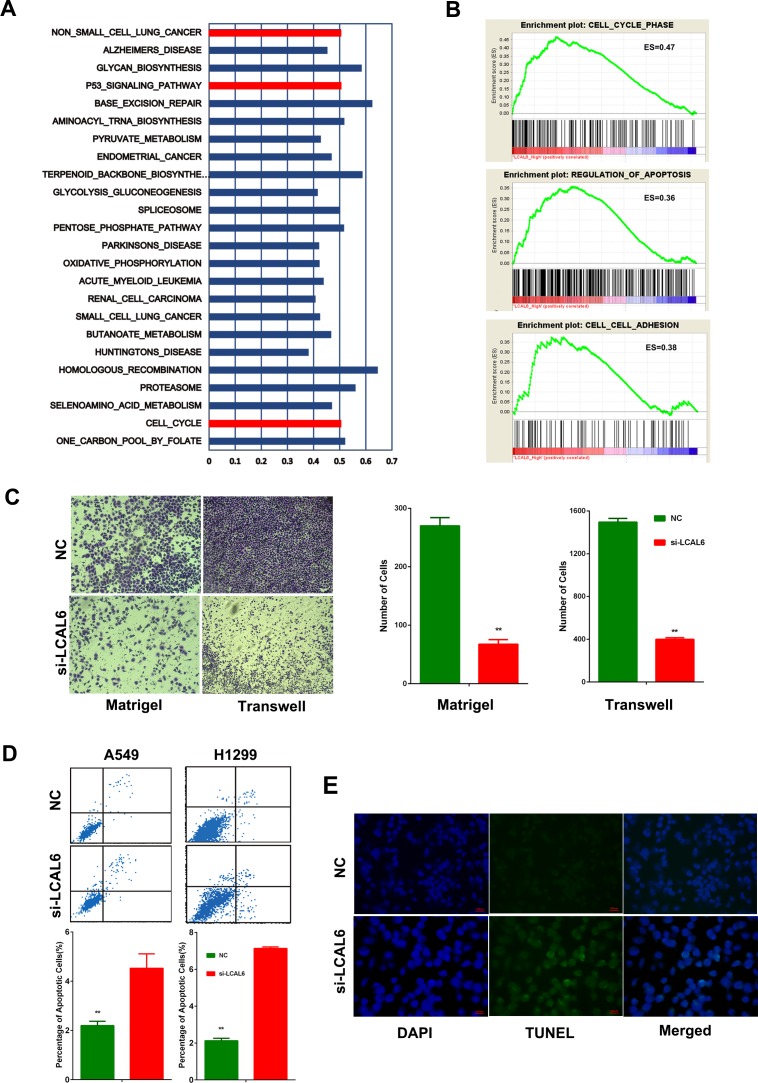
Gene Ontology analyses were performed for protein-coding genes coexpressed with LCAL6 **A.** Gene set enrichment analysis (GSEA) showed that LCAL6 were associated with important cellular function, like cell cycle, apoptosis, and adhesion **B.** Matrigel and transwell assays showed metastasis ability of A549 cell was significantly inhibited by silence of LCAL6 **C.** The percent of apoptotic cells were increased in LUAD cell lines after silence of LCAL6 **D.** as revealed by PI staining. And TUNEL assay confirmed the increased apoptosis in A549 cells.

To further explore the biological function of LCAL6, protein-coding genes those are coexpressed with LCAL6 were identified in the GSE27262 and Gene Ontology (GO) analyses suggested these genes were enriched for items of NSCLC, cell cycle, and P53 signaling pathways (Figure 4A). Gene set enrichment analyses (GSEA) were performed in the GSE27262 dataset and we found LCAL6 was associated with multiple important biological processes, like cell cycle, apoptosis, and cell adhesion (Figure 4B). Accordingly, metastasis ability of LUAD cells also significantly inhibited after silence of LCAL6 as revealed by transwell and matrigel assay. (Figure 4C). And using PI staining and flow cytometry analysis we also found the percentage of apoptotic cells significantly increased after silence of LCAL6 and TUNEL assay confirmed the increased apoptosis in A549 cells (Figure 4D, 4E). Herein, these lines of evidence show that LCAL6 plays an important role in LUAD.

### Predicting function of LCAL6 using lncRNA expression profile

**Figure 5:**
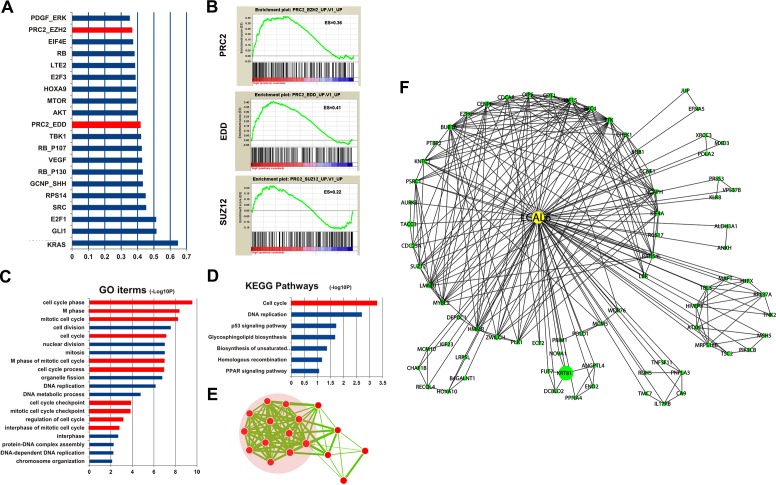
GSEA found PRC2 gene signatures were enriched for LCAL6 **A.**, **B.** Functional annotation found the genes of PRC2 signatures enriched with LCAL6 were associated with cell cycle **C.**, **D.**, **E.** A LCAL6-centered regulatory network was proposed according to GSEA and coexpression analysis **F.**.

It is quite challenging to predict biological functions of lncRNAs. While the expression pattern of protein-coding genes could provide clues to infer biological function of lncRNAs and GSEA is a powerful tool to infer lncRNAs function [30, 31]. GSEA showed that several polycomb repression complex 2 (PRC2) gene signatures were significantly enriched in LUAD samples of high LCAL6 expression level (Figure 5A, 5B). The genes involved in PRC2 signature and enriched in the samples of high LCAL6 expression level were collected and GO functional analyses showed that these genes were significantly associated with cell cycle (Figure 5C, 5D, 5E). PRC2 consists of 3 components, EZH2, SUZ12, and EED, and mediates trimethylation of histone 3 lysine 27 (H3K27). Khalil AM et al and colleagues have reported that approximately 20% of lncRNAs bind to PRC2, indicating that most lncRNAs exert their biological function by binding to RNA-binding proteins, especially PRC2 [32]. In addition, He W et al has reported that LCAL6 could bind with PRC2 and negatively regulate gene expression by modifying trimethylation of H3K27 in bladder cancer [33]. Thus, we propose that LCAL6 could bind with PRC2 and regulate downstream genes (Figure 5F).

## DISCUSSION

In this study, we investigate lncRNA expression profile in LUAD by reannotation the HGU133plus2.0 micararray probe sets. Compared with previous studies, our strategy has several advantages. First, the microarray probe sets were annotated according to the RefSeq database including more than 60,000 lncRNA transcripts and more probe sets mapped to lncRNA transcripts were identified. Secondly, we used the training and validation approach, which improved statistical power and yielded rigorous and stable results. As shown in Supplementary Table S2, several reported lung cancer associated lncRNAs were also found aberrantly expressed in our analyses. Additionally, the aberrant expression signature was also externally validated in a cohort of 20 pairs of LUAD tissues and adjacent non-tumor tissues using qRT-PCR.

Aberrant expression of lncRNAs has been observed in various kinds of diseases and evidence shows that these dysregulated lncRNAs exert vital biological functions [34, 35]. To test the biological function of LCAL6, we designed siRNAs that efficiently silenced LCAL6 expression in 2 LUAD cell lines. As predicted, silence of LCAL6 inhibited proliferation, promoted apoptosis, and suppressed metastasis ability of LUAD cell lines. Furthermore, silence of LCAL6 inhibited LUAD tumor growth *in vivo*, indicating oncogenic potential of LCAL6. In the GSE50081 dataset, high LCAL6 expression level indicated poor survival and LCAL6 expression was positively correlated with tumor size in our qRT-PCR validation. These data proved that LCAL6 promoted LUAD tumor progression and indicated prognosis, suggesting LCAL6 could be a therapeutic target and biomarker.

According to the GENCODE gene annotation V15, there are more than 13000 lncRNAs in the human transcriptome [36], but only a small number of lncRNAs have been clearly characterized. Profile of differentially expressed lncRNAs may guide to search for functional lncRNAs and many lncRNAs are characterized in this way, such as MALAT1 [27], BCAR4 [12], LUADT1 [37]. We identified a list of 856 aberrantly expressed lncRNAs in LUAD, which may shed light on the search of key regulatory lncRNAs or sensitive biomarkers in LUAD. As shown by the pro-oncogenesis capacity of LCAL6, it is highly possible that many of the aberrantly expressed lncRNAs might exert important functions in the carcinogenesis of LUAD. Herein, our results provide valuable resources to identify functional lncRNAs in LUAD

Our study presents a comprehensive analysis of lncRNA in LUAD and we showed an upregulated lncRNA, LCAL6 has vital function in LUAD. These data pave the road for further characterization of functional lncRNAs in LUAD.

## MATERIALS AND METHODS

### Microarray datasets

Microarray datasets were retrieved from the Gene Expression Omnibus (GEO) database. To identify all relevant studies about LUAD expression profiles, we searched GEO database and filtered eligible studies with the following criteria: 1) LUAD tissues were included (>20 samples); 2) normal lung tissues were analyzed for control (>10 samples); 3) the sample microarray platform was used. 4 datasets (GSE19188, GSE19804, GSE27262, and GSE30219) met the selection criteria and were included in our analyses. For GSE19188 and GSE30219, only LUAD and normal samples were retrieved and analyzed. The data meta-analysis processes were shown in Figure 1. Differentially expressed lncRNAs were calculated with the online GEO2R tool (http://www.ncbi.nlm.nih.gov/geo/geo2r/) using adjusted *P* < 0.01 as the threshold.

### lncRNA annotation pipeline

The Affymetrix HG-U133Plus 2.0 microarray was used in the 4 datasets, which includes 54000 probe sets and is widely used in various kinds of biological researches. To identify the probe sets mapped to lncRNAs, we developed a lncRNA annotation pipeline. Step 1, transcripts labeled as “NR_” or “XR_” and larger than 200nt were retrieved from the NCBI Refseq database (60255 lncRNAs were retrieved). The probe sequences of HG-U133Plus 2.0 microarray were also downloaded from the Affymetrix website. Step 2, BLAST software was used to compare the sequences of probe sets and sequences of lncRNAs. If ≥90% sequences of a probe set were matched with a lncRNA, then the probe set was considered as matched with this lncRNA; otherwise, the BLAST result was abandoned. Step 3, the annotation file we achieved in Step 2 was combined with the annotation file by Zhang et al [13]. Therefore, a total of 8068 annotated lncRNA transcripts with corresponding Affymetrix probe IDs were generated (Supplementary Table S6).

### Bioinformatic analyses

Genomic location of 856 differentially expressed lncRNAs were submitted to the website (http://bejerano.stanford.edu/great/public/html/). Gene Ontology (GO) analysis was performed using DAVID website (https://david.ncifcrf.gov/home.jsp). Hierarchical cluster and heat map of differentially expressed lncRNAs were conducted by Cluster 3.0. GSEA was performed by the GSEA software and gene sets used in this work were downloaded from the Molecular Signatures Database (http://software.broadinstitute.org/gsea/msigdb/index.jsp, MSigDB v4.0, released Jun 7, 2013). The MSigDB collects various types of gene set and the online pathway database included 1320 Canonical pathways derived from the pathway databases of BioCarta, KEGG, PID, Reactome and others databases.

### Patients and tissue samples

This study was approved by the Ethics Committee of Cancer Institute of Jiangsu Province. Paired NSCLC tissues and adjacent non-tumor tissues were obtained from 87 patients who received surgical resection of NSCLC between 2012 and 2013 at the department of thoracic surgery, Cancer Institute of Jiangsu Province, China. All surgical specimens were snap-frozen and stored in liquid nitrogen immediately after resection until total RNA extraction. All tumor and paired non-tumor tissues were confirmed by experienced pathologists, as well as the pathological stage, grade, and nodal status. At least 80% tumor samples were composed of viable-appearing tumor cells on histological assessment. Clinical characteristics of analyzed patients were shown in supplementary Table 7. Informed written consents were obtained from all patients included in this study.

### Cell culture and siRNA transfection

All cell lines (A549 and NCI-H1299) were purchased from the Institute of Biochemistry and cell biology of Chinese academy of science (Shanghai, China). A549, and NCI-H1299 cells were cultured in RPMI 1640 medium (KeyGEN, Nanjing, China) supplemented with 10% fetal bovine serum (10% FBS, GIBCO), 100U/ml penicillin, and 100 mg/ml streptomycin (KeyGEN, Nanjing, China) in humidified air at 37°C with 5% CO_2_. A549 and NCI-H1299 cells were transfected with small interfering RNAs (siRNAs) or negative control sequences using Lipofectamine 2000 (Invitrogen, Shanghai, China). siRNA sequences were provided in Supplementary Table S8

### Total RNA extraction and qRT-PCR analysis

Methods of total RNA extraction, reverse transcription, and quantitative real-time polymerase chain reaction (qRT-PCR) have been described before [38]. The PCR primers used were as. The Ct-value for each sample was calculated with the ΔΔCt-method, and the results were expressed as 2^−ΔΔCT^ to analyze the fold change (tumor vs. normal) [38]. qRT-PCR primers were provided in Supplementary Table S8.

### TUNEL assay

A549 and NCI-H1299 cells were seeded on coverslips and 24 hours after transfection with si-LCAL6 or NC sequences, cells were fixed in 4% paraformaldehyde for 15 min at room temperature. Cell apoptosis was detected using Terminal Deoxynucleotidyl Transferase-Mediated dUTP Nick-End Labelling (TUNEL) assay kit (Kaygene, Nanjing, China) according to the manufacturer's instructions. Cells were then washed and stained with DAPI. Coverslips were mounted onto glass slides using a Zeiss Axioscope inverted fluorescence microscope (Zeiss).

### EDU assay

A549 and NCI-H1299 cells were seeded on coverslips and 24 hours after transfection with si-LCAL6 or NC sequences, cells were exposed to 50 μmol/L of 5-ethynyl-20-deoxyuridine (EdU, Ribobio, Guangzhou, China) for additional 4 h at 37°C. Then, cells were fixed with 4% paraformaldehyde for 15 min. EdU incorporation assay was carried out according to the manufacturer's instructions (RiboBio). Cells were then washed and stained with DAPI. Coverslips were mounted onto glass slides using a Zeiss Axioscope inverted fluorescence microscope (Zeiss). EdU, a thymidine analog, incorporation can be used to label cells undergoing DNA replication [39].

### Flow cytometry analysis

Transfected cells were harvested after transfection by trypsinization. After the double staining with fluorescein isothiocyanate (FITC)-Annexin V and propidium iodide was done by the FITC Annexin V Apoptosis Detection Kit (BD Biosciences) according to the manufacturer's recommendations. The cells were analyzed with a flow cytometry (FACScan; BD Biosciences) equipped with a Cell Quest software (BD Biosciences).

### CCK8 assay

NCI-H1975 cell were seeded into 96-well plates (3000/well) and incubated in RPMI 1640 at 37°C and 5% CO_2_ atmosphere for 48 hours. The Cell Counting Kit-8 assay was used to determine relative cell growth according to the manufacturer's instructions. The absorbance was measured at 450 nm with an ELx-800 Universal Microplate Reader. Each experiment was repeated at least three times independently.

### Transwell and matrigel assays

For transwell assay, transfected cells (30000) were plated in the upper chamber of inserts (8 mm pores, Millipore, Billerica, MA) containing 200ul of serum-free 1640 medium. The lower chambers were filled with 500ul 1640 containing 10% FBS. For Matrigel assay, transfected cells (50000) were plated in the top chamber with a matrigel-coated membrane (BD Biosciences) in 400ul serum-free 1640. Also, the bottom chambers were filled with 800ul 1640 containing 10% FBS. After 24 h of incubation for tanswell assay and 48 h for Matrigel assay, the cells on the filter surface were fixed with methanol, stained with crystal violet, and photographed. The number of stained cell was analyzed by Image J software. Each experiment was repeated at least three times independently.

### Xenograft tumor models

A549 cells transfected with si-LCAL6 or negative control (NC) sequence using Lipofectamine 2000 (Invitrogen). After 48 hours of transfection, the cells were collected and injected into either side of the posterior flank of the same male BALB/c nude mouse. The tumor volumes and weights were measured every 4 days in the mice; the tumor volumes were measured as length×width2×0.5. Sixteen days after injection, the mice were sacrificed, the tumor weights were measured, and the tumors were collected for further analysis. The LUADT1 levels were determined by qRT-PCR.

### Immunohistochemistry

Xenograft tumor tissue samples were immunostained for Ki67. Anti-Ki67 antibody was from Santa Cruz Biotechnology.

### Statistical analysis

Student's t-test, one-way ANOVA analysis, linear regression, and Cox regression were performed to analyze the data using SPSS 18.0 software. *P* < 0.05 was considered statistically significant.

## SUPPLEMENTARY FIGURES AND TABLES












