# Supplementary material for: Comprehensive analysis of lncRNA expression profiles and identification of functional lncRNAs in lung adenocarcinoma
Source: Oncotarget. 2016 Feb 21;7(13):16012–22. doi: 10.18632/oncotarget.7559 (PMC4941294; doi:10.18632/oncotarget.7559)
Supplement: Supplementary file 1 [file oncotarget-07-16012-s001.pdf]

Comprehensive analysis of lncRNA expression profiles and identification of functional lncRNAs in lung adenocarcinoma

Supplementary Materials

Table S1. Probe sets mapped to lncRNAs

Table S2. 856 differentially expressed lncRNAs identified

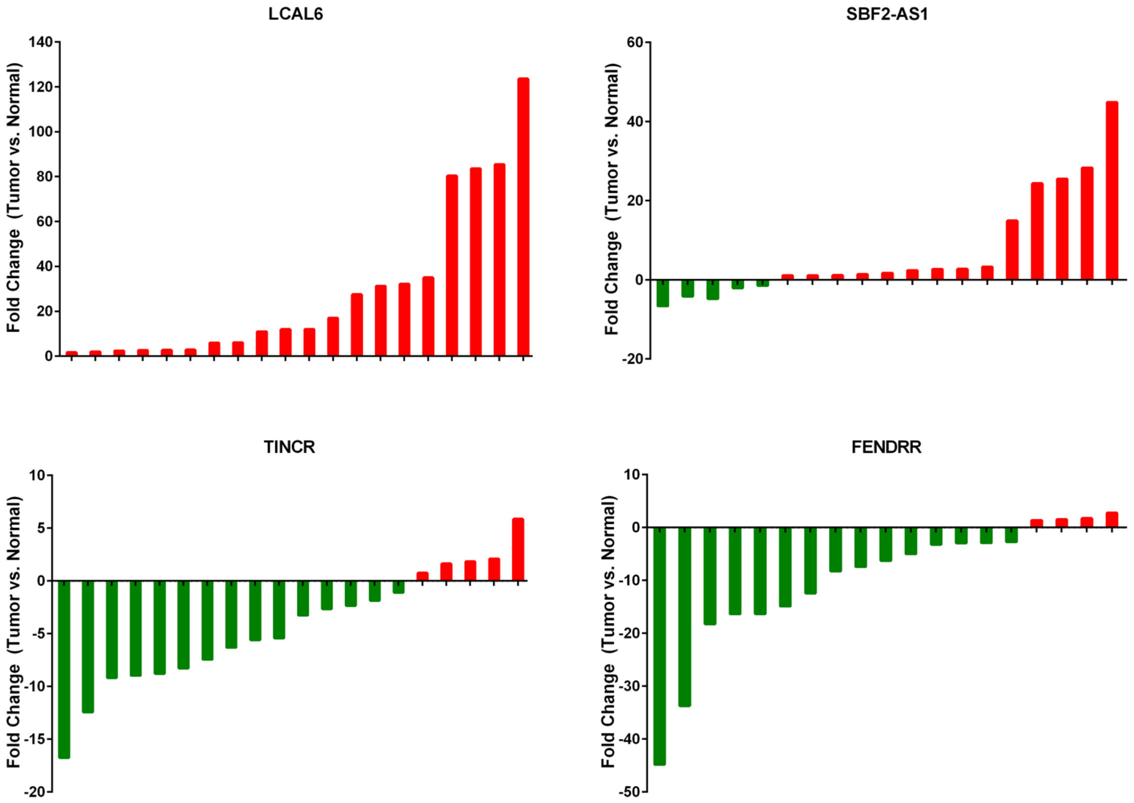

Figure S3. Four lncRNAs were validated by qRT-PCR and regulation patterns of the 4 lncRNAs were consistent with our analyses results.

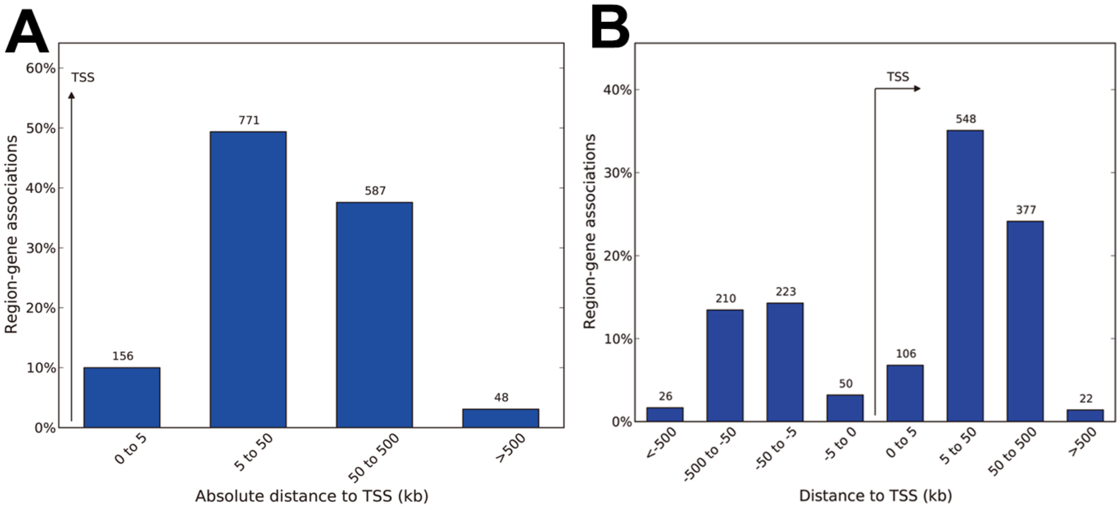

Figure S4. GREAT analysis of the 856 lncRNAs and these lncRNAs are located nearby transcription start sites (A, B).

Table S5. 20 lncRNAs both in our analysis and LCALs

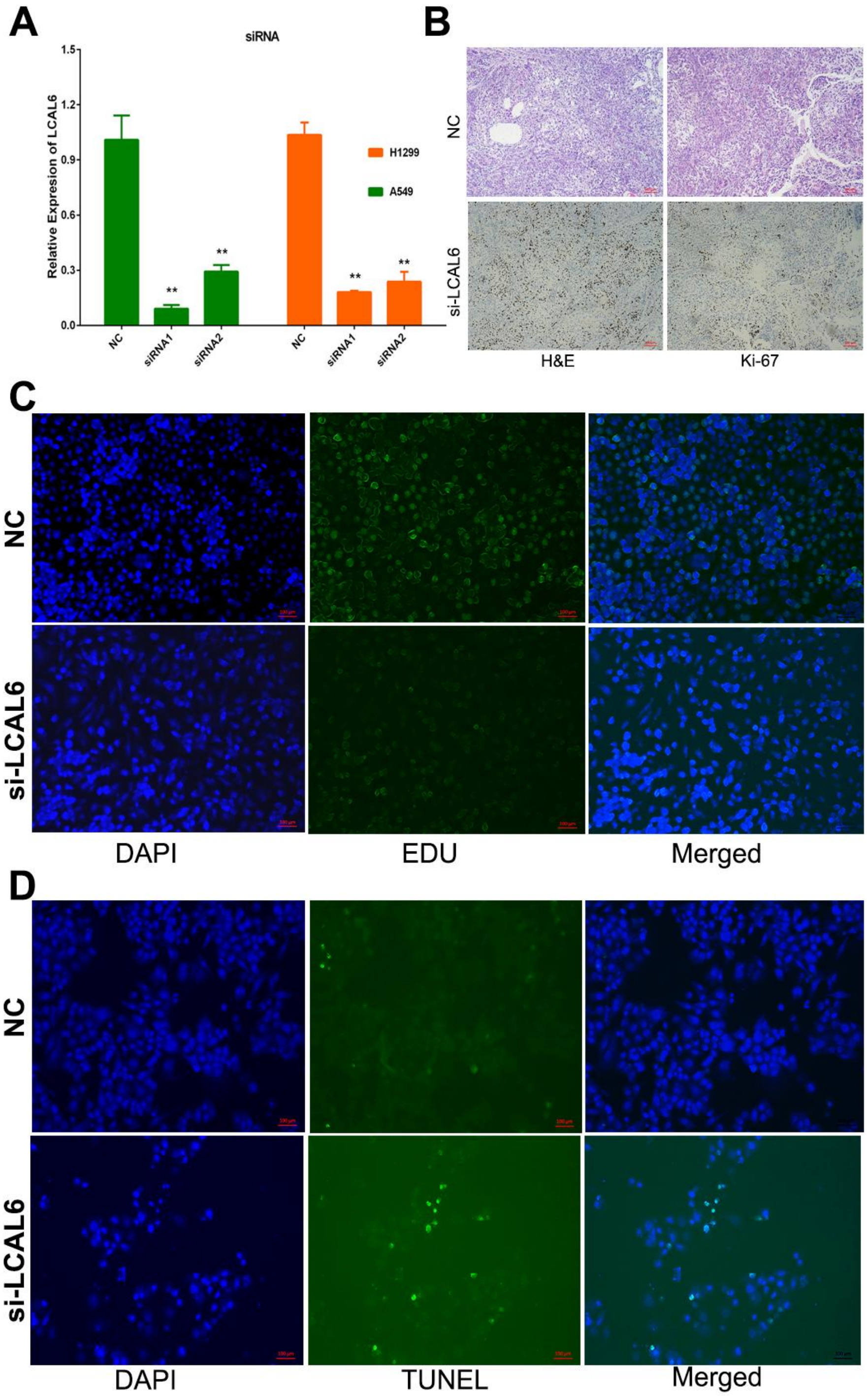

Figure S6. Two siRNAs efficiently silenced LCAL6 in A549 and H1299 cell lines (A).

Immunohistochemistry staining of Ki-67 was weaker in the xenograft tumors derived from A549 cells transfected with si-LCAL6 (B)

EdU assay showed silence of LCAL6 inhibited H1299 cell growth (C), and TUNEL assay showed apoptotic cells increased after silence of LCAL6 in H1299 cells (D).

Table S7. Clinical characteristics of lung cancer patients

Table S8. qRT-PCR primers and siRNA sequences
